# Refining Risk-Stratification of High-Risk and Locoregional Prostate Cancer: A Pooled Analysis of Randomized Trials

**DOI:** 10.1016/j.eururo.2024.04.038

**Published:** 2024-05-22

**Authors:** Praful Ravi, Wanling Xie, Marc Buyse, Susan Halabi, Philip Kantoff, Oliver Sartor, Gert Attard, Noel Clarke, Anthony D’Amico, James Dignam, Nicholas James, Karim Fizazi, Silke Gillessen, Wendy Parulekar, Howard Sandler, Daniel Spratt, Matthew R. Sydes, Bertrand Tombal, Scott Williams, Christopher J. Sweeney

**Affiliations:** 1 Dana-Farber Cancer Institute, Boston, MA, USA; 2 International Drug Development Institute, Louvain-la-Neuve, Belgium; 3 I-BioStat, Hasselt University, Hasselt, Belgium; 4 Duke University, Durham, NC, USA; 5 Convergent Therapeutics, Cambridge, MA, USA; 6 Mayo Clinic, Rochester, MN, USA; 7 University College London, London, UK; 8 The Christie NHS Foundation Trust, Manchester, UK; 9 Brigham & Women’s Hospital, Boston, MA, USA; 10 University of Chicago, Chicago, IL, USA; 11 The Institute of Cancer Research & The Royal Marsden NHS Foundation Trust, London, UK; 12 Institut Gustave Roussy, University of Paris Saclay, Villejuif, France; 13 Oncology Institute of Southern Switzerland, EOC, Bellinzona, Switzerland; 14 Università della Svizzera Italiana, Lugano, Switzerland; 15 Queens University, Kingston, Ontario, Canada; 16 Cedars-Sinai Medical Center, Los Angeles, CA, USA; 17 Case Western Reserve University, Cleveland, OH, USA; 18 Medical Research Council at UCL, London, United Kingdom; 19 Cliniques Universitaires Saint-Luc, Brussels, Belgium; 20 Peter Maccallum Cancer Centre, Melbourne, Australia; 21 South Australian Immunogenomics Cancer Institute, University of Adelaide, Adelaide, Australia

**Keywords:** high-risk prostate cancer, risk stratification, metastasis-free survival, overall survival, radiotherapy, androgen-deprivation therapy

## Abstract

**Background:**

Radiotherapy (RT) and long-term ADT (ltADT; 18-36 months) is a standard-of-care in the treatment of high-risk localized/locoregional prostate cancer (HRLPC). We evaluated outcomes in patients treated with RT + ltADT to identify which patients have poorer prognosis with standard therapy.

**Methods:**

Individual patient data (IPD) from patients with HRLPC (as defined by any of the following 3 risk factors [RFs] in context of cN0 disease: Gleason score ≥8, cT3-T4, PSA >20ng/mL, or cN1) treated with RT and ltADT on randomized controlled trials collated by the Intermediate Clinical Endpoints in Cancer of the Prostate group. Outcome measures of interest were metastasis-free survival (MFS), overall survival (OS), time to metastasis (TTM) and prostate cancer-specific mortality (PCSM). Multivariable Cox and Fine-Gray regression estimated hazard ratios (HR) for the 3 RFs and cN1 disease.

**Findings:**

3604 patients from 10 trials were evaluated, with a median PSA of 24ng/mL. Gleason score ≥8 (MFS HR=1.45; OS HR=1.42), cN1 disease (MFS HR=1.86; OS HR=1.77), cT3-4 disease (MFS: HR=1.28; OS: HR=1.22), and PSA >20ng/mL (MFS HR=1.30; OS HR=1.21) were associated with poorer outcomes. Adjusted 5-year MFS rates were 83% and 78% for patients with 1 and 2-3 RFs, and 10-year MFS rates were 63% and 53%, respectively; corresponding 10-year adjusted OS rates were 67% and 60%. In cN1 patients, adjusted 5- and 10-year MFS rates were 67% and 36%, respectively, and 10-year OS was 47%.

**Conclusion:**

HRLPC patients with 2-3 RFs (and cN0) or cN1 disease had the poorest outcomes on RT and ltADT. This will help in counselling patients treated in routine practice and in guiding adjuvant trials in HRLPC.

## Introduction

Approximately 25% of localized prostate cancers are considered ‘high-risk’, as defined by a Gleason score ≥8 and/or PSA >20ng/mL and/or clinical T3/T4 disease,[1] with evidence of regional nodal involvement seen in an additional 10-15% of cancers.[2] Together, high-risk and locoregional prostate cancer (HRLPC) are associated with a significant risk of prostate cancer mortality and account for two-thirds of deaths from prostate cancer at 10 years.[3]

Multimodal therapy is usually required for HRLPC, with RT and long-term (lt; 18-36 months) androgen deprivation therapy (ADT) being a widely accepted standard-of-care.[4, 5] Recently, the STAMPEDE trial showed a significant improvement in metastasis-free (MFS) and overall survival (OS) with the addition of abiraterone to RT and ltADT in men with HRLPC, as defined by either cN1 disease or two of: Gleason ≥8, cT3-4 and PSA ≥40ng/mL.[6] The STAMPEDE participants represented a particularly high-risk group, with a median PSA of 30-40ng/mL and 40% of patients having N1 disease on conventional imaging. Trials evaluating other novel androgen receptor pathway inhibitors (ARPIs) in combination with RT and ADT for HRLPC are ongoing and are being powered with the assumption of 5-year MFS of ~75% in the control arm of RT + ltADT. (Supplementary Table 1).

Based on these considerations, we sought to evaluate long-term outcomes in various groups of patients with HRLPC treated with RT and ltADT on randomized trials, whose individual patient data (IPD) are available within the Intermediate Clinical Endpoints in Cancer of the Prostate (ICECaP) data repository.[7] Specifically, we aimed to define the outcomes for a range of endpoints – including MFS and OS, but also cancer-specific measures such as time to metastasis (TTM) and prostate cancer-specific mortality (PCSM) – associated with different permutations of standard clinicopathological variables. Defining the patients with HRLPC with the poorest outcomes may help clarify those most likely to benefit from treatment intensification as well as those who may achieve excellent outcomes with RT and ltADT alone and be candidates for treatment de-intensification.

## Methods

### Trial and Patient Selection

The ICECaP repository comprises trials collected in the initial meta-analysis that has been previously published[8] as well as data from additional trials collected between May 2020 and February 2023 since this publication; the meta-analysis was conducted with adherence to PRISMA guidelines. For the current study, only IPD from patients in RT-based trials who had HRLPC and were treated with 18-36 months of ADT were eligible; HRLPC was defined as cN1 disease (on conventional imaging) and/or any of Gleason ≥8, cT3-4 and PSA >20ng/mL. A flowchart of selection of patients for this study is shown in Supplementary Figure 1, and the list of eligible patients from included trials is provided in Supplementary Table 2.

### Definition of endpoints

The clinical outcomes analyzed were MFS, OS, TTM and PCSM. MFS was measured from the date of randomization to date of first evidence of distant metastases (by conventional imaging – CT, MRI and/or bone scan – or histology) or death from any cause; or censored at the date of most recent follow-up. TTM was defined analogously to MFS but non-prostate cancer deaths without prior disease progression were counted as a competing risk. OS was measured from the date of randomization to death from any cause, or censored at the date of most recent follow-up in patients who were alive. PCSM was defined similarly as OS, but non-prostate deaths were considered as a competing risk.

### Statistical Analysis

5-year MFS and OS were estimated by the Kaplan Meier method; 5-year of TTM and PCSM were estimated using cumulative incidence function accounting for competing risk. Multivariable Cox regression models (for MFS and OS) and the Fine and Gray Competing risks regression (for TTM and PCSM) were performed to estimate the strength of association of clinical outcomes with pre-defined baseline risk factors, including biopsy Gleason (≥8 vs. ≤7), clinical T-stage (cT3-4 vs. cTx1-2), PSA at randomization (<10ng/mL, 10-20ng/mL, and >20ng/mL), and clinical N-stage (cN1 vs. cN0). The PSA cutoffs were based on established risk stratification criteria for localized prostate cancer.[9] These models were adjusted for age at randomization, ADT duration (≥24 months vs. 18 months) and radiotherapy dose (≤70Gy, >70Gy and unknown) and stratified by years of enrollment (per 5-year increment) to account for variability of follow up times across the trials. Median follow-up was calculated using the reverse Kaplan-Meier method.

Based on number of baseline adverse risk factors from the multivariable models above, we estimated adjusted 5- and 10-year MFS and OS from Cox regression[10] and adjusted 5- and 10-year TTM and PCSM[11] from Fine and Gray regression models. Additionally, we reported unadjusted Kaplan Meier estimates of MFS and OS and unadjusted cumulative incidence of TTM and PCSM for various pre-planned risk subgroups (by permutations of Gleason, clinical T-stage, PSA and clinical N stage) as well as for post-hoc analyses of number of adverse factors by age (≤ vs >68 years) and radiotherapy dose delivered (≤70Gy, >70Gy and unknown) using the median as a threshold for each stratification variable. The adjusted survival curves were estimated using R “adjustedCurve” package (https://www.r-project.org/). All other statistical analyses were performed using the SAS software application (version 9.4; SAS Institute, Cary, NC, USA). Two-sided p values <0.05 were considered statistically significant.

## Results

A total of 3604 patients with HRLPC treated across 10 trials evaluating RT and ltADT were eligible. Baseline characteristics of these patients at the time of randomization are shown in Table 1. Median age was 68 years and median PSA was 24ng/mL; 1942 patients (54%) had Gleason 8-10 disease, 2061 (57%) had a PSA >20ng/mL, 2602 (72%) were cT3-4, and 422 (12%) had cN1 disease. Median follow-up was 8.6 years (interquartile range 6.0-11.8), and 5-year MFS and OS rates in the entire population were 78% (95% CI 77-80) and 84% (83-85), respectively.

Table 2 shows the results of multivariable analyses evaluating the adjusted associations of clinical risk factors with long-term outcomes. Statistically significant associations were seen for Gleason score ≥8 (MFS HR=1.45 [95% CI 1.29-1.63]; OS HR=1.42 [1.26-1.61]), cN1 disease (MFS HR=1.86 [1.56-2.21]; OS HR=1.77 [1.45-2.15]), cT3-4 disease (MFS HR=1.28 [1.13-1.45]; OS HR=1.22 [1.07-1.39]), and PSA >20ng/mL (MFS HR=1.30 [1.13-1.50]; OS HR=1.21 [1.05-1.41]). Broadly similar trends were seen in the associations between these variables and TTM and PCSM.

Given the variability in associations between the clinicopathological variables and outcomes, we generated Kaplan-Meier estimates of 5- and 10-year MFS rates based on various permutations of risk factors (Gleason 7 vs ≥8, PSA <10 vs 10-20 vs ≥20ng/mL, cT3-4 vs cTx1-2, cN1; Table 3); estimates of 5- and 10-year OS, TTM and PCSM are shown in Supplementary Table 3. Overall, outcomes were best in cN0 patients with just one adverse risk factor (Gleason ≥8, PSA >20ng/mL, cT3-4), intermediate in patients with 2 adverse risk factors and worse in patients with all 3 risk factors; the poorest outcomes overall were seen in patients with cN1 disease regardless of other risk factors.

**Figure 1:**
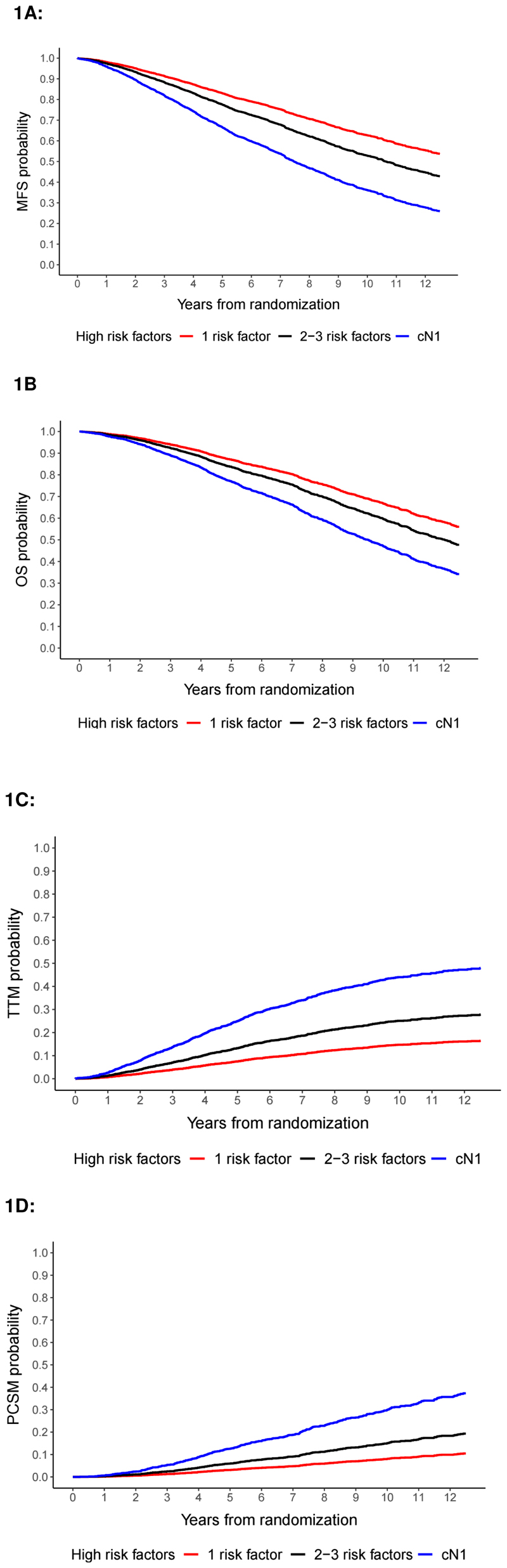
Adjusted curves showing MFS (2A) and OS (2B) from Cox regression models and TTM (2C) and PCSM (2D) from the Fine and Gray models, based on number of adverse baseline risk factors (Gleason ≥8, cT3-4 and PSA >20ng/mL) or cN1 disease. All models were adjusted for age at randomization, ADT duration (≥24 months vs 18 months) and radiotherapy dose (≤70 Gy, >70 Gy and unknown). Abbreviations: MFS – metastasis-free survival; TTM – time to metastasis; OS – overall survival; PCSM – prostate cancer-specific mortality

Given the similar outcomes between cN0 patients with 2 or 3 adverse risk factors, these were grouped together and adjusted survival curves showing MFS and OS, and cumulative incidence of TTM and PCSM based on number of risk factors (1 vs. 2-3 vs. cN1) are shown in Figure 1. Adjusted 5- and 10-year estimates of MFS, OS, TTM and PCSM rates by these risk groups (1 vs. 2-3 vs. cN1) are shown in Table 4. Adjusted 5-year MFS rates were 83% (81-85), 78% (76-79) and 67% (62-71) for patients with 1, 2-3 risk factors and cN1 disease, respectively, while corresponding adjusted 5-year OS rates were 87% (86-88), 84% (82-85) and 77% (74-80). Similar trends in outcomes by risk groups were seen when stratifying by age or RT dose (Supplementary Tables 4-5), with generally better outcomes seen across risk groups in patients treated at higher RT doses.

We also evaluated the STAMPEDE definition of high-risk in our cohort (i.e. cN1 or Gleason 8-10, cT3-4, PSA ≥40ng/mL), which led to a decrease in the number of patients with 2-3 risk factors. Despite the higher PSA cut-off, very similar adjusted 5- and 10-year outcomes were observed within each risk group (1 vs 2-3 vs cN1) when using either STAMPEDE or conventional criteria (Supplementary Table 6, Supplementary Figure 2).

## Discussion

In this analysis comprising 3604 patients treated on 10 randomized trials of RT and ltADT for HRLPC, we noted statistically significant and clinically meaningful differences in long-term outcomes based on the overall number of baseline adverse risk factors. Specifically, patients with at least two risk factors (Gleason 8-10, cT3-4, PSA >20ng/mL) in context of cN0 disease, or cN1 disease (regardless of other risk factors) had poorer outcomes compared to those with only 1 risk factor, with a 5-year MFS of 78% for cN0 patients with 2-3 risk factors and 67% for all patients with cN1 disease, versus 83% for patients with 1 risk factor and cN0. Moreover, the number of prostate cancer events contributing to the MFS and OS endpoints increased with the poorer risk groups, indicating that those patients more likely to develop life-threatening clinical events are potentially more likely to benefit from treatment intensification beyond RT and ltADT.

Since D’Amico and colleagues developed the first risk classification scheme for localized prostate cancer in the late 1990s,[12] the presence of biopsy Gleason 8-10, cT3-T4 and/or PSA >20ng/mL at diagnosis have been taken forward by guideline groups, such as EAU[4], ESMO[13] and NCCN[9], to define high-risk disease. However, outcomes within this group are heterogeneous and there have been subsequent efforts to refine risk stratification[14–17]. These have typically used these three variables to generate prognostic groups that are better able to risk-stratify patients, but have been limited by evaluation of patients undergoing surgery (and not RT and ADT), heterogeneity in treatments received and lack of significant numbers of patients receiving ltADT with RT. As such, our findings represent the largest study to define risk stratification within HRLPC, are the first to evaluate patients receiving ADT in addition to RT, use IPD from randomized trials, and corroborate these earlier efforts that a simple assessment of the number of risk factors (1 vs 2-3 vs N1) can provide more robust prognostic information.

These results have several important implications for clinical practice as well as in the interpretation of ongoing (neo)adjuvant trials in HRLPC. The addition of 2 years of abiraterone to RT and ltADT has become a standard-of-care for “very” high-risk M0 prostate cancer based on the STAMPEDE-abiraterone trial.[6] That comparison of the STAMPEDE study comprised of ~40% N1 patients (by conventional imaging), with the remainder having two of Gleason 8-10, cT3-4 or PSA ≥40ng/mL, and the median PSA in the trial was 30-40ng/mL. In our analyses, very similar results in long-term outcomes were seen when patients were classified by either EAU/ESMO/NCCN high-risk criteria or STAMPEDE high-risk criteria. As such, N0 patients with 2 or 3 adverse risk factors (by EAU/ESMO/NCCN criteria) had a 5-year MFS <80% with RT and ltADT, and likely to benefit from the addition of abiraterone. In contrast, N0 patients with just one high-risk factor had better long-term outcomes with RT and ltADT, whereas N1 patients denoted a particularly high-risk group in whom intensification might be of greatest benefit.

There are several ongoing adjuvant trials assessing the addition of other ARPIs to RT and ltADT in HRLPC. Eligibility criteria vary between these trials, with baseline data from the ATLAS,[18] ENZARAD AND DASL trials[19] showing a range in cN1 disease from 11-28% and a median PSA in the ATLAS trial of 6ng/mL, which are notably different to the STAMPEDE population. Our results will be helpful to provide a framework upon which to guide clinical decision-making, based on extent of risk factors and by N0 vs N1 disease, thereby guiding the interpretation of these studies.

It is important to note that none of the patients included in our analysis had molecular imaging (e.g. PSMA-PET) for staging or evaluation of suspected recurrence or metastasis. PSMA-PET has greater sensitivity, specificity and diagnostic accuracy compared to conventional imaging in staging high-risk disease.[20] As such, our findings and outcome estimates only apply to those with high-risk and/or N1 disease on conventional scans, which is reflected in the 5-year MFS rate of 80% amongst high-risk N0 patients treated with RT and ltADT. This is lower than the 5-year MFS of 89% in patients with N0 disease treated with prostate-only RT and ltADT in the POP-RT trial, where the median PSA was similar to our cohort (28ng/mL vs. 24ng/mL), but 80% of patients were staged with PSMA-PET.[21] This indicates that the absence of nodal disease on PET is highly prognostic. As such, it is to be determined whether high-risk patients with one risk factor and <1cm PSMA-avid pelvic nodes (i.e. N0 by conventional imaging) would benefit from intensification of therapy beyond whole pelvis RT and ltADT alone.

The strengths of this work lie in the availability of IPD from multiple randomized trials with a median follow-up of nearly 9 years ensuring that the 5- and 10-year MFS estimates we provide are robust and can serve as a benchmark for ongoing trials and in counselling patients treated in routine practice. We specifically chose not to evaluate PSA-based endpoints, such as biochemical failure or event-free survival, since these have not shown to be good surrogates for OS.[22, 23] While there are other efforts ongoing to define which people may benefit most from addition of ADT (and beyond) to RT in high-risk disease,[24] the risk stratification we provide is based on inexpensive, readily available parameters that are already routinely used in everyday practice.

Despite these, we acknowledge key limitations, including the long time period over which trial participants were treated (1987-2016), lack of data on therapies utilized at recurrence, lack of data on the actual ADT duration that patients received, and heterogeneity in RT field, dose and fractionation, though we noted better outcomes amongst patients treated at RT doses of >70Gy (i.e. above the median) of this cohort, in line with recent data from the GETUG-AFU 18 study[25]. Nevertheless, we adjusted for RT dose and planned ADT duration as well as stratifying by years of enrolment in our multivariate analyses. We additionally lacked information on whether T staging was assigned by imaging or digital rectal exam (DRE), and outcomes might be better in those with radiologic T3-T4 disease only. Molecular imaging was not used in staging (or monitoring) patients, and studies are needed to define how PSMA-PET imaging can improve upon the data defined by clinicopathological variables and conventional imaging.

In summary, this IPD analysis comprising approximately 3600 patients treated with RT and ltADT for HRLPC demonstrated important prognostic differences between patients depending on the presence of specific risk factors (Gleason 8-10, cT3-4, PSA >20ng/mL; cN1), alone or in combination. Patients with 2-3 risk factors (in the context of cN0 disease) or cN1 disease (regardless of other risk factors) had 5-year MFS rates of <80% and appear to be the best candidates for intensification of therapy beyond RT and ltADT. These findings have implications for selection of patients for therapy intensification in clinical practice, and will be helpful in interpreting the results of ongoing adjuvant studies in HRLPC.

## Supplementary Material

Supp 1

## Figures and Tables

**Table 1:** Baseline characteristics at randomization of included patients

	N (%)
Age, yrs, median (IQR)	68 (63-73)
Year of randomization	
1987-1994	724 (20)
1995-1999	256 (7.1)
2000-2004	768 (21)
2005-2009	850 (24)
2010-2016	1006 (28)
PSA at randomization, ng/mL, median (IQR)	24 (12-48)
<10	719 (20)
10-20	806 (22)
>20	2061 (57)
Unknown	18 (0.50)
Biopsy Gleason score	
<7	564 (16)
7	1069 (30)
8-10	1942 (54)
Unknown	29 (0.80)
Clinical T stage	
Tx1-2	1002 (28)
T3-4	2602 (72)
Clinical N1	422 (12)
Planed duration of ADT treatment	
18 months	365 (10)
≥24 months	3239 (90)
Radiotherapy dose, Gy, median (IQR)*	70 (69-74)

* evaluable N=2990

Abbreviations: ADT-Androgen Deprivation Therapy; IQR – interquartile range

**Table 2:** Multivariable models estimating the associations between long-term outcomes and baseline clinical parameters

	MFS		OS		TTM		PCSM	
	HR (95% CI)	p	HR (95% CI)	p	sHR (95% CI)	p	sHR (95% CI)	p
Biopsy Gleason ≥8 (ref: ≤7)	1.45(1.29-1.63)	<.001	1.42(1.26-1.61)	<.001	1.84 (1.55-2.19)	<.001	2.08(1.66-2.60)	<.001
Clinical T3-4 (ref: Tx1-2)	1.28(1.13-1.45)	<.001	1.22(1.07-1.39)	0.003	1.58(1.30-1.91)	<.001	1.73(1.35-2.22)	<.001
PSA at randomization (ref: <10)								
10-20ng/mL	1.08(0.92-1.27)	0.4	1.05(0.89-1.25)	0.5	1.06(0.84-1.34)	0.6	1.03(0.77-1.38)	0.9
>20ng/mL	1.30(1.13-1.50)	<.001	1.21(1.05-1.41)	0.011	1.30(1.06-1.59)	0.011	1.05(0.82-1.36)	0.7
Clinical N1 (ref: N0)	1.86(1.56-2.21)	<.001	1.77(1.45-2.15)	<.001	2.17(1.73-2.73)	<.001	2.43(1.79-3.30)	<.001
Age at randomization (per year)	1.02(1.01-1.03)	<.001	1.04(1.03-1.05)	<.001	0.97(0.96-0.98)	<.001	0.97(0.96-0.99)	0.001
Radiotherapy dose (ref: d70 Gy)								
>70 Gy	1.05(0.91-1.22)	0.5	1.00(0.86-1.17)	>0.9	0.96(0.78-1.18)	0.7	0.73(0.55-0.97)	0.032
Unknown	1.42(1.17-1.74)	0.001	1.35(1.09-1.68)	0.006	1.30(0.98-1.74)	0.071	1.19(0.82-1.74)	0.4
ADT ≥24 months (ref: 18 months)	0.80(0.64-0.99)	0.039	0.93(0.74-1.18)	0.6	0.61(0.45-0.81)	0.001	0.73(0.49-1.08)	0.11

Abbreviations: ADT – androgen deprivation therapy; MFS – metastasis-free survival; OS – overall survival; TTM – time to metastasis; PCSM – prostate cancer-specific mortality; HR – hazard ratio; sHR – subdistribution hazard ratio; CI – confidence interval

**Table 3:** Unadjusted Kaplan Meier estimates of 5-year and 10-year MFS rates (95% CI) in various subgroups of patients, stratified by risk factors (Gleason score, PSA, cT stage; and cN1) at baseline. NB – all patients with cN1 disease were analyzed together and stratified by Gleason score at diagnosis.

	*Gleason 7*		*Gleason 8-10*	
	*Tx1-2*	*T3-4*	*Tx1-2*	*T3-4*
**5-year MFS**				
*PSA <10ng/mL*	*-*	87 (82-91)	82 (76-87)	75 (69-80)
*PSA 10-20ng/mL*	*-*	81 (75-85)	84 (77-89)	79 (73-83)
*PSA >20ng/mL*	84 (79-87)	80 (76-83)	74 (67-79)	77 (73-80)
*cN1*	76 (67-82)		64 (58-69)	
**10-year MFS**				
*PSA <10ng/mL*		65 (57-72)	62 (54-68)	52 (43-60)
*PSA 10-20ng/mL*		57 (50-64)	63 (54-70)	59 (51-66)
*PSA >20ng/mL*	63 (57-68)	59 (54-64)	47 (39-54)	46 (40-52)
*cN1*	36 (200-53)		38 (28-47)	

Abbreviations: MFS – metastasis-free survival; CI – confidence interval

**Table 4:** Adjusted estimates of 5-year and 10-year MFS and OS from Cox regression and TTM and PCSM from the Fine and Gray models, based on number of baseline adverse risk factors (Gleason 8-10, cT3-4, PSA >20ng/mL) and cN1 disease. All models were adjusted for age at randomization, ADT duration (≥24 months vs 18 months) and radiotherapy dose (≤70 Gy, >70 Gy and unknown).

	N	No. of events	5-year % (95% CI)	10-year % (95% CI)
**MFS**				
1 risk factor	1241	508	83(81-85)	63(60-66)
2-3 risk factors	1900	796	78(76-79)	53(50-56)
cN1	422	188	67(62-71)	36(31-42)
**OS**				
1 risk factor	1241	467	87(86-88)	67(64-70)
2-3 risk factors	1900	683	84(82-85)	60(57-62)
cN1	422	144	77(74-80)	47(41-53)
**TTM**				
1 risk factor	1241	184	7.5(6.3-8.8)	15(13-17)
2-3 risk factors	1900	400	13(12-15)	25(23-28)
cN1	422	137	25(21-29)	44(38-50)
**PCSM**				
1 risk factor	1241	106	3.1(2.4-3.8)	8.0(6.6-9.6)
2-3 risk factors	1900	237	5.9(5.0-7.0)	15(13-17)
cN1	422	78	13(10-16)	30(25-35)

Abbreviations: MFS – metastasis-free survival; OS – overall survival; TTM – time to metastasis; PCSM – prostate cancer-specific mortality; CI – confidence interval
