# Supplementary material for: Refining Risk-Stratification of High-Risk and Locoregional Prostate Cancer: A Pooled Analysis of Randomized Trials
Source: Eur Urol. Author manuscript; Available in PMC 2025 Feb 25. (PMC11579255; doi:10.1016/j.eururo.2024.04.038)
Supplement: Supp 1 [file EMS202888-supplement-Supp_1.docx]

**SUPPLEMENTARY MATERIAL**

**Supplementary Table 1 – Ongoing adjuvant trials in high-risk/locally advanced prostate cancer**

| **Trial (NCT)** | **N** | **Comparison** | **Eligibility** | **Results/Primary Endpoint** |
| --- | --- | --- | --- | --- |
| STAMPEDE (NCT00268476) | 1974 | Trial 1: ltADT vs ltADT + abiraterone  Trial 2: ltADT vs ltADT + abiraterone + enzalutamide | Either cN1 by conventional imaging or at least two of the following: a) ≥cT3 b) Gleason 8-10 c) PSA ≥40 ng/mL | MFS  Trial 1: HR=0.54 (0.43-0.68)  Trial 2: HR=0.53 (0.39-0.71) |
| ATLAS (NCT02531516) | 1503 | ltADT vs ltADT + apalutamide | 1 of the following: a) Gleason ≥8 and ≥cT2c b) Gleason ≥7, PSA ≥20 ng/mL, and ≥cT2c | MFS |
| ENZARAD (NCT02446444) | 802 | ltADT vs ltADT + enzalutamide | 1 of the following: a) Gleason 8-10  b) Gleason 4+3 and ≥cT2b-4 and PSA ≥20ng/mL c) cN1 by conventional imaging | MFS |
| DASL-HiCAP (NCT04136353) | 1100 | ltADT vs ltADT + darolutamide | 1 of the following: a) Gleason 9-10  b) Gleason 8 and any of the following:  - ≥cT2b  - MRI with T3a/T3b disease   - PSA ≥20ng/mL c) cN1 by conventional imaging | MFS |
| PREDICT-RT (NCT04513717) | 786 | ltADT vs ltADT + apalutamide | Intensification arm: cN1 by conventional imaging, or Decipher >0.85 with 1 of the following:  a) PSA >20ng/mL  b) ≥cT3  c) Gleason 8-10 | MFS |
| PEACE-2 (NCT01952223) | 1048 | Prostate +/- pelvic RT + ltADT +/- cabazitaxel | At least 2 of:  a) Gleason 8-10  b) cT3-4  c) PSA ≥20ng/mL | cPFS |

Abbreviations: ltADT – long-term ADT; RT – radiotherapy; cPFS – clinical progression-free survival

**Supplementary Table 2 – Trials included in the analysis**

| **Study** | **Year Enrolled** | **Arm** | **Treatment** | **RT dose, median (range)** | **Total N** | **Eligible N** |
| --- | --- | --- | --- | --- | --- | --- |
| EORTC 22863^1^ | 1987-1995 | Experimental | RT+ AADT 3yr | 70 (18-90) | 415 | 204 |
| EORTC 22961^2^ | 1997-2001 | Experimental | RT + NADT 6mo + AADT 2.5yr | 70 (64-74) | 970 | 436 |
| French study (Mottet)^3^ | 2000-2003 | Experimental | RT + ADT 3yr | 70 (65-76) | 264 | 129 |
| GICOR-DART 01/05^4^ | 2005-2010 | Experimental | RT + NADT 4mo + AADT 2yr | 78 (31-83) | 352 | 91 |
| RTOG 9202^5^ | 1992-1995 | Experimental | RT + NADT 4mo + AADT 2yr | 68 (13-77) | 1520 | 617 |
| GETUG 12^6^ | 2002-2006 | Control | RT + AADT 3yr | 74 (69-80) | 413 | 204 |
| RTOG 0521^7^ | 2005-2009 | Control | RT + AADT 2yr | NA | 563 | 281 |
| RTOG 9902^8^ | 2000-2004 | Control | RT + AADT 2yr | NA | 397 | 197 |
| STAMPEDE^9-12^ | 2006-2016 | Control | RT + AADT ≥2yr | 74 (8.0-156) | 2537 | 1080* |
| TROG 0304^13^ | 2003-2007 | Experimental | RT + NADT 6mo + AADT 12mo | 70 (46-76) | 1071 | 365 |
| Total |  |  |  |  | 8502 | 3604 |

*In the STEMPEDE trial, there were 2142 controls from 1080 unique subjects for 7 treatment comparisons. If the same subject was used as multiple controls, data with longest follow-up was retained for individual patient level analysis.

Abbreviations: AADT – adjuvant ADT; NADT – neoadjuvant ADT; RT – radiotherapy; NA - Not available

References: ^1^ Bolla et al, Lancet 2002; ^2^ Bolla et al, NEJM 2009; ^3^ Mottet et al, Eur Urol 2012; ^4^ Zapatero et al, Lancet Oncol 2015; ^5^ Hanks et al, J Clin Oncol 2003; ^6^ Fizazi et al, Lancet 2015; ^7^ Rosenthal et al, J Clin Oncol 2019; ^8^ Rosenthal et al, Int J Radiat Oncol Biol Phys 2015**;** ^9^ James et al, Lancet 2016; ^10^ James et al, JNCI Cancer Spect 2022; ^11^ Mason et al, J Clin Oncol 2017; ^12^ Attard et al, Lancet 2022; ^13^ Denham et al, Lancet Oncol 2019

**Supplementary Table 3A – Unadjusted Kaplan Meier estimates of MFS and OS at 5- and 10-years in various risk subgroups**

| **Group** | **Total N** | **MFS** | | | **OS** | | |
| --- | --- | --- | --- | --- | --- | --- | --- |
|  |  | **No. of events** | **5-year % (95% CI)** | **10-year % (95% CI)** | **No. of events** | **5-year % (95% CI)** | **10-year % (95% CI)** |
| GS≤7 T3-4 PSA<10 | 232 | 81 | 87 (82-91) | 65 (57-72) | 77 | 88 (82-91) | 68 (60-75) |
| GS≤7 T3-4 PSA 10-20 | 286 | 127 | 81 (75-85) | 57 (50-64) | 120 | 84 (79-88) | 64 (57-70) |
| GS≤7 Tx1-2 PSA>20 | 364 | 162 | 84 (79-87) | 63 (57-68) | 143 | 89 (85-92) | 68 (62-73) |
| GS≤7 T3-4 PSA>20 | 612 | 253 | 80 (76-83) | 59 (54-64) | 221 | 84 (81-87) | 66 (61-70) |
| GS ≤7 cN1 | 130 | 55 | 76 (67-82) | 36 (20-53) | 45 | 83 (75-88) | 51 (33-66) |
| GS≥8 Tx1-2 PSA<10 | 195 | 74 | 82 (76-87) | 62 (54-68) | 70 | 89 (83-92) | 64 (56-71) |
| GS≥8 Tx1-2 PSA 10-20 | 164 | 64 | 84 (77-89) | 63 (54-70) | 57 | 89 (83-93) | 68 (59-75) |
| GS≥8 T3-4 PSA<10 | 231 | 95 | 75 (69-80) | 52 (43-60) | 79 | 82 (76-86) | 59 (50-67) |
| GS≥8 T3-4 PSA 10-20 | 267 | 99 | 79 (73-83) | 59 (51-66) | 87 | 84 (79-88) | 65 (57-72) |
| GS≥8 Tx1-2 PSA>20 | 220 | 116 | 74 (67-79) | 47 (39-54) | 104 | 84 (79-89) | 53 (45-60) |
| GS≥8 T3-4 PSA>20 | 570 | 233 | 77 (73-80) | 46 (40-52) | 192 | 83 (79-86) | 55 (49-61) |
| GS ≥8, cN1 | 289 | 132 | 64 (58-69) | 38 (28-47) | 98 | 76 (71-81) | 46 (34-56) |

Abbreviations: GS – Gleason score; MFS – metastasis-free survival; OS – overall survival

**Supplementary Table 3B – Unadjusted cumulative Incidence of TTM and PCSM at 5- and 10-years in various risk subgroups from competing risk models**

| **Group** | **Total N** | **TTM** | | | **PCSM** | | |
| --- | --- | --- | --- | --- | --- | --- | --- |
|  |  | **No. of events** | **5-year % (95% CI)** | **10-year % (95% CI)** | **No. of events** | **5-year % (95% CI)** | **10-year % (95% CI)** |
| GS≤7 T3-4 PSA<10 | 232 | 20 | 4.1 (2.0-7.4) | 9.3 (5.7-14) | 15 | 3.3 (1.5-6.5) | 7.0 (3.7-12) |
| GS≤7 T3-4 PSA 10-20 | 286 | 42 | 7.6 (4.8-11) | 17 (12-22) | 27 | 3.2 (1.6-5.8) | 7.9 (4.8-12) |
| GS≤7 Tx1-2 PSA>20 | 364 | 60 | 7.1 (4.8-10) | 16 (12-20) | 23 | 1.4 (0.54-3.1) | 6.3 (3.9-9.5) |
| GS≤7 T3-4 PSA>20 | 612 | 100 | 7.8 (5.8-10) | 18 (14-22) | 55 | 3.6 (2.3-5.3) | 8.9 (6.3-12) |
| GS ≤7 cN1 | 130 | 38 | 19 (13-26) | 46 (28-62) | 25 | 11 (6.3-17) | 31 (16-47) |
| GS≥8 Tx1-2 PSA<10 | 195 | 35 | 11 (6.8-16) | 19 (13-25) | 24 | 3.8 (1.7-7.3) | 13 (8.4-19) |
| GS≥8 Tx1-2 PSA 10-20 | 164 | 27 | 8.9 (5.1-14) | 18 (12-25) | 17 | 3.3 (1.2-7.0) | 11 (6.4-17) |
| GS≥8 T3-4 PSA<10 | 231 | 58 | 18 (13-23) | 27 (21-34) | 35 | 10 (6.8-15) | 17 (11-23) |
| GS≥8 T3-4 PSA 10-20 | 267 | 56 | 14 (10-19) | 24 (18-31) | 41 | 7.9 (5.0-12) | 18 (13-24) |
| GS≥8 Tx1-2 PSA>20 | 220 | 49 | 14 (10-19) | 26 (20-32) | 33 | 3.8 (1.8-7.0) | 18 (12-24) |
| GS≥8 T3-4 PSA>20 | 570 | 137 | 15 (12-18) | 33 (28-38) | 73 | 6.7 (4.8-9.0) | 19 (15-24) |
| GS ≥8, cN1 | 289 | 98 | 28 (23-33) | 44 (35-53) | 52 | 12 (8.8-16) | 29 (21-38) |

TTM – time to metastasis; PCSM – prostate cancer-specific mortality

**Supplementary Table 4 – Unadjusted estimates of 5-year and 10-year MFS, OS, TTM and PCSM based on number of baseline adverse risk factors (Gleason 8-10, cT3-4, PSA >20ng/mL) and cN1 disease, by age groups.**

|  | **Age ≤68 years (median)** | | | | **Age >68 years (median)** | | | |
| --- | --- | --- | --- | --- | --- | --- | --- | --- |
|  | **N** | **No. of events** | **5-year % (95% CI)** | **10-year % (95% CI)** | **N** | **No. of events** | **5-year % (95% CI)** | **10-year % (95% CI)** |
| **MFS*** |  |  |  |  |  |  |  |  |
| 1 risk factor | 611 | 215 | 85 (82-88) | 67 (63-71) | 629 | 293 | 81 (78-84) | 56 (51-60) |
| 2-3 risk factors | 948 | 360 | 80 (77-83) | 57 (52-61) | 952 | 436 | 75 (72-78) | 49 (45-54) |
| cN1 | 257 | 112 | 66 (60-72) | 39 (27-51) | 165 | 76 | 70 (62-76) | 31 (18-45) |
| **OS*** |  |  |  |  |  |  |  |  |
| 1 risk factor | 611 | 185 | 91 (88-93) | 74 (69-77) | 629 | 282 | 84 (81-87) | 59 (54-63) |
| 2-3 risk factors | 948 | 288 | 87 (84-89) | 66 (61-69) | 952 | 395 | 80 (77-83) | 55 (51-59) |
| cN1 | 257 | 80 | 79 (74-84) | 52 (39-64) | 165 | 64 | 77 (69-83) | 37 (22-51) |
| **TTM**** |  |  |  |  |  |  |  |  |
| 1 risk factor | 611 | 107 | 8.5 (6.4-11) | 18 (15-22) | 629 | 77 | 6.5 (4.7-8.7) | 13 (10-16) |
| 2-3 risk factors | 948 | 236 | 14 (12-16) | 30 (27-34) | 952 | 164 | 12 (10-14) | 20 (17-23) |
| cN1 | 257 | 92 | 29 (23-35) | 49 (37-60) | 165 | 45 | 19 (14-26) | 41 (28-54) |
| **PCSM**** |  |  |  |  |  |  |  |  |
| 1 risk factor | 611 | 59 | 2.6 (1.5-4.1) | 9.2 (6.8-12) | 629 | 47 | 3.0 (1.9-4.7) | 8.0 (5.8-11) |
| 2-3 risk factors | 948 | 136 | 6.1 (4.7-7.8) | 18 (15-21) | 952 | 101 | 5.8 (4.4-7.5) | 12 (9.8-15) |
| cN1 | 257 | 52 | 13 (9.0-17) | 32 (21-44) | 165 | 26 | 10 (6.3-16) | 29 (17-42) |

***** Kaplan-Meier estimates; ** Cumulative incidence from competing risk models

Abbreviations: MFS – metastasis-free survival; OS – overall survival; TTM – time to metastasis; PCSM – prostate cancer-specific mortality; CI – confidence interval

**Supplementary** **Table 5 – Unadjusted estimates of 5-year and 10-year MFS, OS, TTM and PCSM based on number of baseline adverse risk factors (Gleason 8-10, cT3-4, PSA >20ng/mL) and cN1 disease, by radiotherapy dose delivered.**

|  | **Radiation dose ≤ 70 Gy** | | | | **Radiation dose > 70 Gy** | | | |
| --- | --- | --- | --- | --- | --- | --- | --- | --- |
|  | **N** | **No. of events** | **5-year % (95% CI)** | **10-year % (95% CI)** | **N** | **No. of events** | **5-year % (95% CI)** | **10-year % (95% CI)** |
| **MFS*** |  |  |  |  |  |  |  |  |
| 1 risk factor | 659 | 297 | 83 (80-86) | 59 (55-63) | 282 | 109 | 83 (78-87) | 63 (56-69) |
| 2-3 risk factors | 807 | 398 | 75 (72-78) | 51 (47-55) | 817 | 262 | 83 (80-85) | 57 (51-62) |
| cN1 | 94 | 48 | 74 (64-82) | 41 (26-55) | 291 | 119 | 68 (62-73) | 39 (27-51) |
| **OS*** |  |  |  |  |  |  |  |  |
| 1 risk factor | 659 | 280 | 87 (84-90) | 63 (58-67) | 282 | 98 | 88 (83-91) | 69 (62-74) |
| 2-3 risk factors | 807 | 363 | 80 (77-83) | 57 (52-61) | 817 | 204 | 88 (86-90) | 66 (60-70) |
| cN1 | 94 | 44 | 83 (73-89) | 49 (34-63) | 291 | 84 | 79 (74-83) | 49 (35-61) |
| **TTM**** |  |  |  |  |  |  |  |  |
| 1 risk factor | 659 | 97 | 7.1 (5.2-9.2) | 15 (12-19) | 282 | 42 | 8.7 (5.7-13) | 16 (12-22) |
| 2-3 risk factors | 807 | 180 | 13 (11-16) | 25 (21-28) | 817 | 143 | 11 (8.8-13) | 23 (19-28) |
| cN1 | 94 | 31 | 21 (13-30) | 41 (27-55) | 291 | 89 | 25 (20-30) | 44 (32-55) |
| **PCSM**** |  |  |  |  |  |  |  |  |
| 1 risk factor | 659 | 58 | 2.5 (1.5-4.0) | 9.5 (7.1-12) | 282 | 25 | 3.1 (1.4-5.7) | 8.1 (4.9-12) |
| 2-3 risk factors | 807 | 122 | 7.1 (5.4-9.0) | 16 (13-19) | 817 | 64 | 4.3 (3.0-5.8) | 12 (8.6-15) |
| cN1 | 94 | 23 | 9.8 (4.8-17) | 31 (19-44) | 291 | 43 | 10 (7.2-14) | 26 (17-37) |

***** Kaplan-Meier estimates; ** Cumulative incidence from competing risk models

Abbreviations: MFS – metastasis-free survival; OS – overall survival; TTM – time to metastasis; PCSM – prostate cancer-specific mortality; CI – confidence interval

**Supplementary Table 6 – Adjusted estimates of 5-year and 10-year MFS and OS from Cox regression and TTM and PCSM from the Fine and Gray model, based on number of baseline adverse risk factors by the STAMPEDE criteria (Gleason 8-10, cT3-4, PSA ≥40ng/mL) and cN1 disease**. All models were adjusted for age at randomization, ADT duration (≥24 vs 18 months) and radiotherapy dose (≤70 Gy, >70 Gy and unknown).

|  | **N** | **No. of events** | **5-year % (95% CI)** | **10-year % (95% CI)** |
| --- | --- | --- | --- | --- |
| **MFS** | | | | |
| 1 risk factor | 1582 | 684 | 82(80-84) | 61(58-63) |
| 2-3 risk factors | 1559 | 620 | 77(76-79) | 53(50-56) |
| cN1 | 422 | 188 | 67(62-71) | 36(31-42) |
| **OS** | | | | |
| 1 risk factor | 1582 | 628 | 86(85-88) | 65(62-68) |
| 2-3 risk factors | 1559 | 522 | 84(82-85) | 60(57-63) |
| cN1 | 422 | 144 | 77(74-80) | 47(41-53) |
| **TTM** | | | | |
| 1 risk factor | 1582 | 241 | 7.7(6.6-8.7) | 15(13-17) |
| 2-3 risk factors | 1559 | 343 | 14(13-16) | 27(25-30) |
| cN1 | 422 | 137 | 25(21-29) | 44(39-51) |
| **PCSM** | | | | |
| 1 risk factor | 1582 | 142 | 3.2(2.6-3.9) | 8.4(7.1-9.8) |
| 2-3 risk factors | 1559 | 201 | 6.5(5.5-7.6) | 16(14-19) |
| cN1 | 422 | 78 | 13(10-16) | 30(24-36) |

Abbreviations: MFS – metastasis-free survival; OS – overall survival; TTM – time to metastasis; PCSM – prostate cancer-specific mortality; CI – confidence interval

**Supplementary Figure 1 – Flowchart of selection of patients and trials included in the analysis**

31 RT-based trials in ICECaP repository (n=25,374)

10 trials evaluating RT + ltADT (n=8502)

Patients with HR/LA-PC treated with RT + ltADT (n=3606)

21 trials evaluating other treatments (n=16,872)

Patients without HR/LA-PC or assigned to therapy other than RT + ltADT (n=4898)

Abbreviations: RT – radiotherapy; ltADT – long-term ADT; HR/LA-PC – high-risk and/or locally-advanced prostate cancer

**Supplementary Figure 2 –Adjusted curves showing MFS (A) and OS (B) from Cox regression models and TTM (C) and PCSM (D) from the Fine and Gray models, based on number of adverse baseline risk factors by the STAMPEDE high-risk criteria (Gleason ≥8, cT3-4 and PSA ≥40 ng/mL) or cN1 disease. All models were adjusted for age at randomization, ADT duration (≥24 vs 18 months) and radiotherapy dose (≤70 Gy, >70 Gy and unknown).**

| (A)  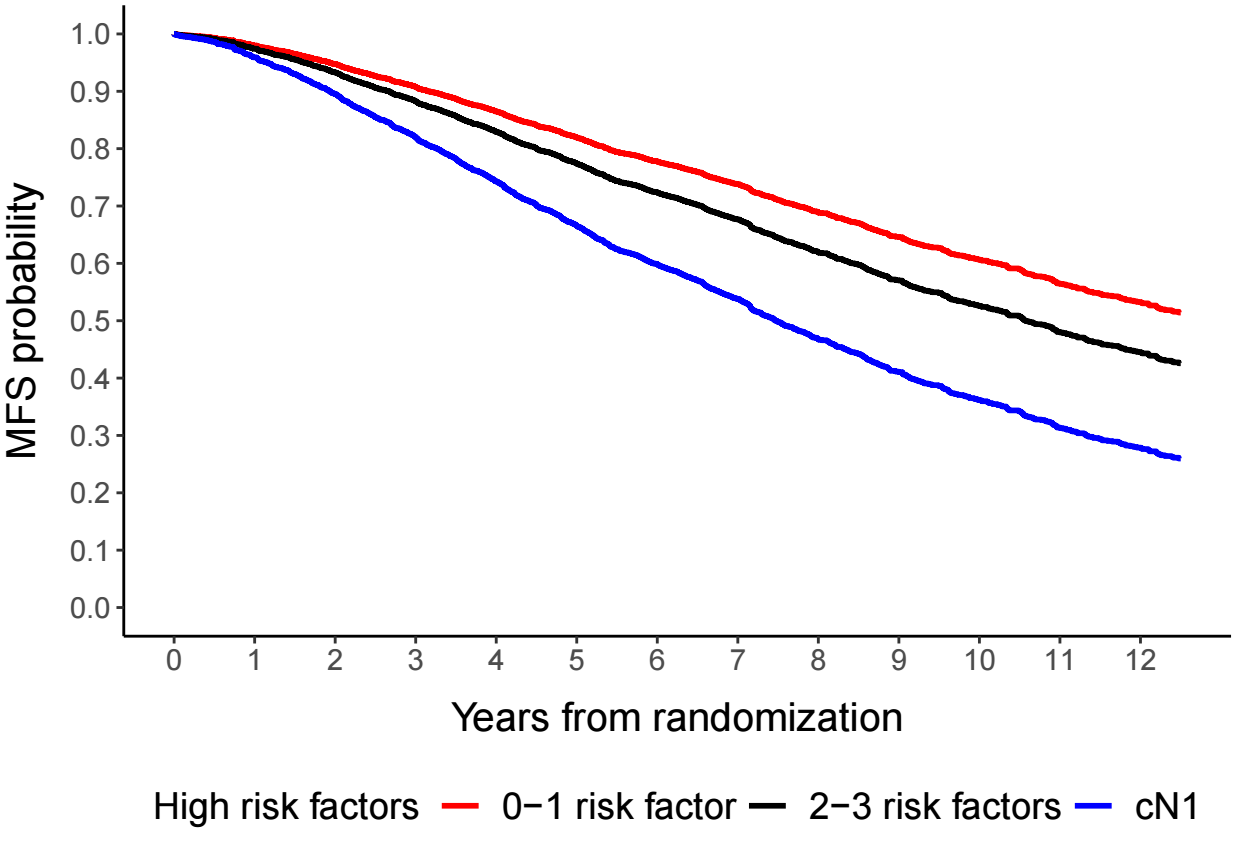 | (B)  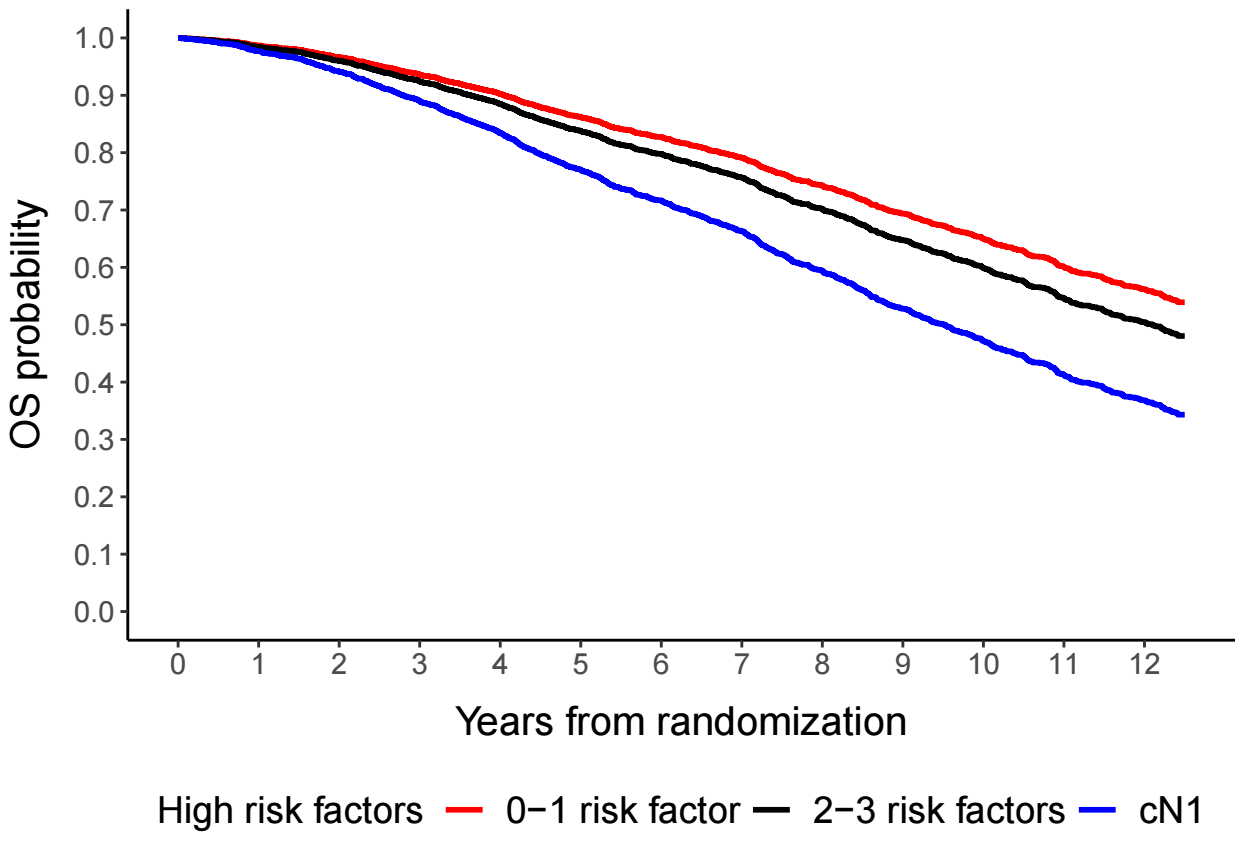 |
| --- | --- |

| (C)  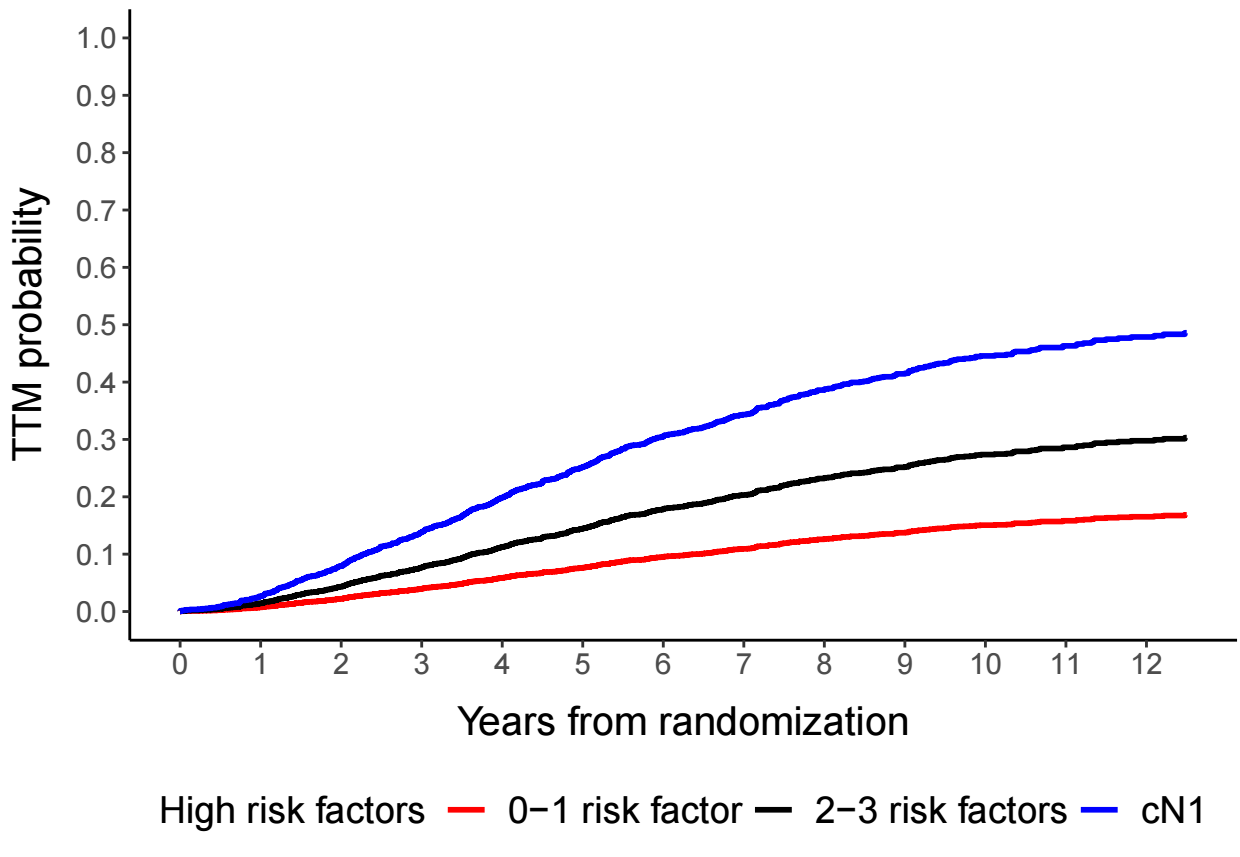 | (D)  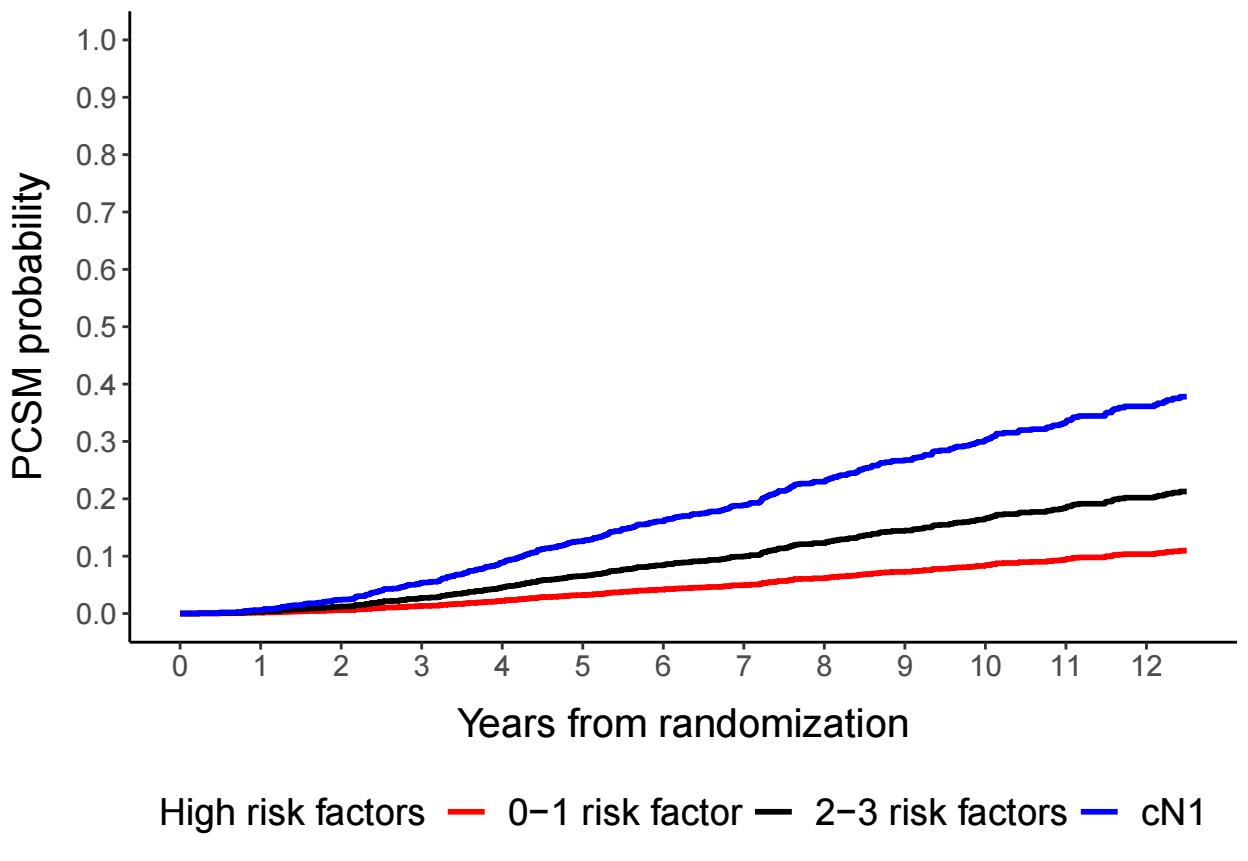 |
| --- | --- |
